# Identification of drug resistance mutations among *Mycobacterium bovis* lineages in the Americas

**DOI:** 10.1371/journal.pntd.0009145

**Published:** 2021-02-16

**Authors:** Carlos Arturo Vázquez-Chacón, Felipe de Jesús Rodríguez-Gaxiola, Cruz Fernando López-Carrera, Mayra Cruz-Rivera, Armando Martínez-Guarneros, Ricardo Parra-Unda, Eliakym Arámbula-Meraz, Salvador Fonseca-Coronado, Gilberto Vaughan, Paúl Alexis López-Durán

**Affiliations:** 1 Facultad de Medicina y Cirugía, Universidad Autónoma Benito Juárez de Oaxaca, Oaxaca, México; 2 Laboratorio de Micobacterias, Instituto de Diagnóstico y Referencia Epidemiológicos, Ciudad de México, México; 3 Facultad de Estudios Superiores Cuautitlán, Universidad Nacional Autónoma de México, Estado de México, México; 4 Escuela Nacional de Ciencias Biológicas, Instituto Politécnico Nacional, Ciudad de México, México; 5 Facultad de Medicina, Universidad Nacional Autónoma de México, Ciudad de México, México; 6 Unidad de Investigaciones en Salud Pública, Facultad de Ciencias Químico Biológicas, Universidad Autónoma de Sinaloa, Culiacán, Sinaloa, México; 7 Laboratorio de Genética y Biología Molecular, Facultad de Ciencias Químico Biológicas, Universidad Autónoma de Sinaloa, Culiacán, Sinaloa, México; 8 Facultad de Ciencias de la Salud, Universidad Anáhuac, Campus Norte, Estado de México, México; Faculty of Science, Ain Shams University (ASU), EGYPT

## Abstract

Identifying the *Mycobacterium tuberculosis* resistance mutation patterns is of the utmost importance to assure proper patient’s management and devising of control programs aimed to limit spread of disease. Zoonotic *Mycobacterium bovis* infection still represents a threat to human health, particularly in dairy production regions. Routinary, molecular characterization of *M*. *bovis* is performed primarily by spoligotyping and mycobacterial interspersed repetitive units (MIRU) while next generation sequencing (NGS) approaches are often performed by reference laboratories. However, spoligotyping and MIRU methodologies lack the resolution required for the fine characterization of tuberculosis isolates, particularly in outbreak settings. In conjunction with sophisticated bioinformatic algorithms, whole genome sequencing (WGS) analysis is becoming the method of choice for advanced genetic characterization of tuberculosis isolates. WGS provides valuable information on drug resistance and compensatory mutations that other technologies cannot assess. Here, we performed an analysis of the most frequently identified mutations associated with tuberculosis drug resistance and their genetic relationship among 2,074 *Mycobacterium bovis* WGS recovered primarily from non-human hosts. Full-length gene sequences harboring drug resistant associated mutations and their phylogenetic relationships were analyzed. The results showed that *M*. *bovis* isolates harbor mutations conferring resistance to both first- and second-line antibiotics. Mutations conferring resistance for isoniazid, fluoroquinolones, streptomycin, and aminoglycosides were identified among animal strains. Our findings highlight the importance of molecular surveillance to monitor the emergence of mutations associated with multi and extensive drug resistance in livestock and other non-human mammals.

## Author summary

Here we describe the identification of high confidence mutations among *Mycobacterium bovis* lineages associated with resistance to first- and second-line antituberculosis drugs. Resistance to isoniazid, aminoglycosides and fluoroquinolones among non-human hosts is of importance because of the risk of emergence of multi- and extensively-drug resistance. Further research is warranted for the identification of the mechanisms responsible for acquisition of such mutations as well as the routes of transmissions involved in this process, including selective use of antibiotics or anthroponotic transmission. Our findings highlight the importance of molecular surveillance to monitor the emergence or introduction of strains carrying mutations related to MDR and XDR. This could potentially help to limit the transmission of pathogenic and difficult to treat TB.

## Introduction

Tuberculosis (TB) is a public health issue worldwide. *Mycobacterium tuberculosis* affects ~1.7 billion people including 1.2 million deaths annually [1]. The emergence of drug resistant lineages, particularly multi- (MDR) and extensively- (XDR) drug resistance is particularly worrisome due to the implications for patients’ management [2]. Drug resistance is primarily due to genetic changes in the bacterial genome, with few exceptions such as phenotypic drug tolerance [3].

The *Mycobacterium tuberculosis* complex (MTC) is a genetically related group of *Mycobacterium* species that can cause tuberculosis in humans and other mammals [4]. MTC includes several variants (vars); *M*. *tuberculosis*, *M*. *bovis*, *M*. *africanum*, *M*. *caprae*, *M*. *microti*, *M*. *pinnipedii*, *M*. *canetti*, *M*. *mungi* and *M*. *orygis* [5]

*M*. *bovis* is the etiologic agent of bovine tuberculosis, a chronic disease of animals, causing economic losses in endemic regions [6]. *M*. *bovis* primarily affects cattle but can also infect humans (zoonotic transmission) and in some cases it might result in MDR and XDR TB [7–10]. Human infection is primarily associated with ingestion of contaminated, unpasteurized milk or dairy products and by direct contact with infected animals [11]. Importantly, *M*. *bovis* accounts for ~1.4% of human tuberculosis cases annually; which makes it a worrisome public health issue [12]. Understanding the phylodynamics and phylogeography of members of the MTC is the key to monitor the circulation of lineages and introduction of novel strains in a given region. Timely identification of MDR and XDR strains is critical for patient management, implementation of control measures as well as for containment of outbreaks.

The arrival of a plethora of next generation sequencing technologies has greatly facilitated the advanced characterization of mycobacterial strains in different settings as well as the identification of mutations conferring drug resistance. Likewise, whole genome sequencing (WGS) strategies for outbreak investigations, molecular epidemiology and surveillance have significantly advanced our understanding on mycobacteria transmissions [13]. These novel approaches can also provide insights on biomarkers associated with traits such as virulence and tropism [14,15]. Here, we performed a comprehensive analysis aimed to identify high confidence drug resistance mutation among 2,074 *M*. *bovis* isolates primarily recovered from non-human hosts. We also carried out a phylogenetic analysis to determine the genetic relationships among these isolates.

## Methods

### Whole genome sequences

*Mycobacterium bovis* WGS (2,074 sequences) were recovered from NCBI database SRA by SRAToolkit using the following criteria: *Mycobacterium bovis*, America, USDA. WGS belonged to livestock (84.7%), and other non-human mammal hosts (15.3%) including 20 human strains (1%). Sequences with a minimum of 40X depth of coverage were included in the study. The vSNP (https://github.com/USDA-VS/vSNP) pipeline was used to establish genetic relatedness among different strains [16–18]. Reads were aligned to the reference genome *M*. *bovis* AF2122/97 [19] NCBI accession number NC_0002945, using Burrows-Wheller Alignment (BWA). Single nucleotide polymorphisms (SNPs) were identified using Freebayes and reported as variant call format (VCF) files [20,21]. Results were filtered using a minimum QUAL score of 150 and alternate allele call (AC) of = 2, with a minimum SNP coverage of 20X. VCF profiles of closely related samples were grouped, SNPs were validated and filtered, to generate the corresponding aligned FASTA files. Maximum likelihood phylogenetic trees were created with RaxML using GTR-CAT as a substitution model [22]. Tree visualization was performed with iTOL v.5.6.2 (itol.embl.de).

### Characterization of drug resistant mutations

Full-length genes sequences (*ald*a (cycloserine), *ald*A (cycloserine) *ald*b (cycloserine), *ald*C (cycloserine), *alr* (cycloserine), *atp*C (bedaquiline), *atp*E (bedaquiline, clofazimine), *ddn* (delamanid), *emb*B (ethambutol), *eth*A (ethionamide, prothionamide), *fab*G1 (isoniazid), *fbi*A (delamanid), *gyr*A (fluoroquinolones), *gyr*B (fluoroquinolones), *had*A (isoxyl, thiacetazone), *had*B (isoxyl, thiacetazone), *inh*A (isoniazid), *ini*C (isoniazid), *kas*A (isoniazid), *kat*G (isoniazid), *pan*D (pyrazinamide), *pep*Q (bedaquiline, clofazimine), *pnc*A (pyrazinamide), *rpl*C (linezolid), *rpl*D (linezolid), *rpo*B (rifampicin), *rps*L (streptomycin), *rrl* (linezolid), *rrs* (aminoglycosides, streptomycin), *tly*A (capreomycin), BQ2027_MB0697 (bedaquiline, clofazimine), BQ2027_MB2001C (bedaquiline, clofazimine), BQ2027_MB2022C (ethionamide), BQ2027_MB3950C (streptomycin) [*Rv0678*, *Rv1979c*, *Rv1999c*, *Rv3919c* orthologues of *M. tuberculosis* H37Rv respectively]) were extracted, aligned, translated to amino acids and mapped to the reference genome. High confidence mutations conferring drug resistance were identified as previously reported [23].

## Results

### Resistance-related mutations

*M*. *bovis* infection has been neglected particularly when occurring in non-human hosts. Here, we seek to identify all high confidence mutations conferring resistance to first- and second-line antibiotics among *M*. *bovis* strains recovered from the Americas and available from publicly accessible databases. The data set included 2,074 different strains primarily recovered from bovine, although 20 human strains were also included in the study (S1 Table). High confidence resistance-associated mutations observed among these isolates are summarized in table 1. Forty-nine strains (2.3%) harbored high confidence resistance-related mutations. Strain SRR7236341 exhibited two drug resistance-related mutations against aminoglycosides and streptomycin, a1401g nucleotide change in the *rrs* gene, as well as the *rps*L K43R mutation. Isoniazid resistant mutations located in the *kat*G gene, GTC → GGG, S315T (Ser^315^Thr), were found in two strains SRR7240297 and SRR7240428. Streptomycin associated mutations in the *rps*L gene, AAG → AGG, K43R (Lys^43^Arg) and AAG → AGG, K88R (Lys^88^Arg), were found in twenty-six and in eight different strains, respectively. Finally, mutations associated with resistance to fluoroquinolones in *gyr*A GAC → GGC, D94G (Asp^94^Gly), were found among 13 strains within the Quinolone Resistance Determining Region (QRDR) [24].

**Table 1:** High-confidence mutations associated with resistance to first- and second-line drugs in *M*. *tuberculosis* found in *M*. *bovis*.

SRA	Strain ID	Strain Origin	Collection Date	NT Change	NT Position	AA Change one letter	AA Change	AA Position	Phylogenetic Group
*kat*G (isoniazid)									
SRR7240297	94–4159	NA	1994	agc/acc	944	S/T	Ser/Thr	315	10
SRR7240428	94–2346	NA	1994	agc/acc	944	S/T	Ser/Thr	315	10
*rps*L (streptomycin)									
SRR1791723	01–2757	USA, CO	2001	aag/agg	128	K/R	Lys/Arg	43	7
SRR1791907	04–2378	MEXICO	2004	aag/agg	128	K/R	Lys/Arg	43	17
SRR1792194	09–5047	USA, TX	2009	aag/agg	128	K/R	Lys/Arg	43	17
SRR1792195	09–5050	USA, TX	2009	aag/agg	128	K/R	Lys/Arg	43	17
SRR1792196	09–5187	USA, TX	2009	aag/agg	128	K/R	Lys/Arg	43	17
SRR1792197	09–5188	USA, TX	2009	aag/agg	128	K/R	Lys/Arg	43	17
SRR1792198	09–5189	USA, TX	2009	aag/agg	128	K/R	Lys/Arg	43	17
SRR1792308	11–8257	NA	2001	aag/agg	128	K/R	Lys/Arg	43	7
SRR1792435	13–5449	NA	2013	aag/agg	128	K/R	Lys/Arg	43	22
SRR7236114	97 1525	MEX, QRO	1997	aag/agg	128	K/R	Lys/Arg	43	17
SRR7236119	09–0305	MEX, EDO MEX	2009	aag/agg	128	K/R	Lys/Arg	43	7
SRR7236133	97 2410	MEX, QRO	1997	aag/agg	128	K/R	Lys/Arg	43	7
SRR7236195	09–0491	MEX, COAH	2009	aag/agg	128	K/R	Lys/Arg	43	4
SRR7236205	97 2171	MEX, QRO	1997	aag/agg	128	K/R	Lys/Arg	43	7
SRR7236218	10–0296	MEX, EDO MEX	2010	aag/agg	128	K/R	Lys/Arg	43	7
SRR7236263	09–0328	MEX, EDO MEX	2009	aag/agg	128	K/R	Lys/Arg	43	23
SRR7236276	97 2453	MEX, QRO	1997	aag/agg	128	K/R	Lys/Arg	43	17
SRR7236341	09–0213	MEX, JAL	2009	aag/agg	128	K/R	Lys/Arg	43	7
SRR7236343	09–0219	MEX, JAL	2009	aag/agg	128	K/R	Lys/Arg	43	23
SRR7236349	09–0418	EDO MEX	2009	aag/agg	128	K/R	Lys/Arg	43	7
SRR7236363	14–805568	MEX, QRO	2014	aag/agg	128	K/R	Lys/Arg	43	17
SRR7236385	09–0278	MEX, JAL	2009	aag/agg	128	K/R	Lys/Arg	43	7
SRR7236386	09–0257	MEX, JAL	2009	aag/agg	128	K/R	Lys/Arg	43	23
SRR7240158	95–2382	NA	1995	aag/agg	128	K/R	Lys/Arg	43	17
SRR7240159	95–2449	NA	1995	aag/agg	128	K/R	Lys/Arg	43	22
SRR7240209	92–5113	NA	1992	aag/agg	128	K/R	Lys/Arg	43	7
SRR1792489	95–2601	USA, MI	1995	aag/agg	263	K/R	Lys/Arg	88	16
SRR7236327	97–2268	MEX, QRO	1997	aag/agg	263	K/R	Lys/Arg	88	16
SRR7240041	92–6735	NA	1992	aag/agg	263	K/R	Lys/Arg	88	16
SRR7240042	93–2054	NA	1993	aag/agg	263	K/R	Lys/Arg	88	16
SRR7240043	93–2159	NA	1993	aag/agg	263	K/R	Lys/Arg	88	16
SRR7240049	93–2248	NA	1993	aag/agg	263	K/R	Lys/Arg	88	16
SRR7240050	93–2304	NA	1993	aag/agg	263	K/R	Lys/Arg	88	16
SRR7240422	93–2409	NA	1993	aag/agg	263	K/R	Lys/Arg	88	16
*gyr*A (fluoroquinolones)									
SRR1792311	12–1962	USA, CA	2012	gac/ggc	281	D/G	Asp/Gly	94	6
SRR1792312	12–0119	USA, CA	2012	gac/ggc	281	D/G	Asp/Gly	94	6
SRR1792314	12–0217	USA, CA	2012	gac/ggc	281	D/G	Asp/Gly	94	6
SRR1792315	12–0221	USA, CA	2012	gac/ggc	281	D/G	Asp/Gly	94	6
SRR1792316	12–0241	USA, CA	2012	gac/ggc	281	D/G	Asp/Gly	94	6
SRR1792317	12–0251	USA, CA	2012	gac/ggc	281	D/G	Asp/Gly	94	6
SRR1792318	12–0257	USA, CA	2012	gac/ggc	281	D/G	Asp/Gly	94	6
SRR1792320	12–0812	USA, CA	2012	gac/ggc	281	D/G	Asp/Gly	94	6
SRR1792326	12–1278	USA, CA	2012	gac/ggc	281	D/G	Asp/Gly	94	6
SRR1792327	12–1601	USA, CA	2012	gac/ggc	281	D/G	Asp/Gly	94	6
SRR1792332	12–3012	USA, CA	2012	gac/ggc	281	D/G	Asp/Gly	94	6
SRR1792337	12–4832	USA, CA	2012	gac/ggc	281	D/G	Asp/Gly	94	6
SRR1792342	12–5103	USA, CA	2012	gac/ggc	281	D/G	Asp/Gly	94	6
*rrs* (aminoglycosides)									
SRR7236341	09–0213	MEX, JAL	2009	a/g	1401	-	-	-	7

NA = Not available

### Genetic relatedness among resistant mutants

**Fig 1:**
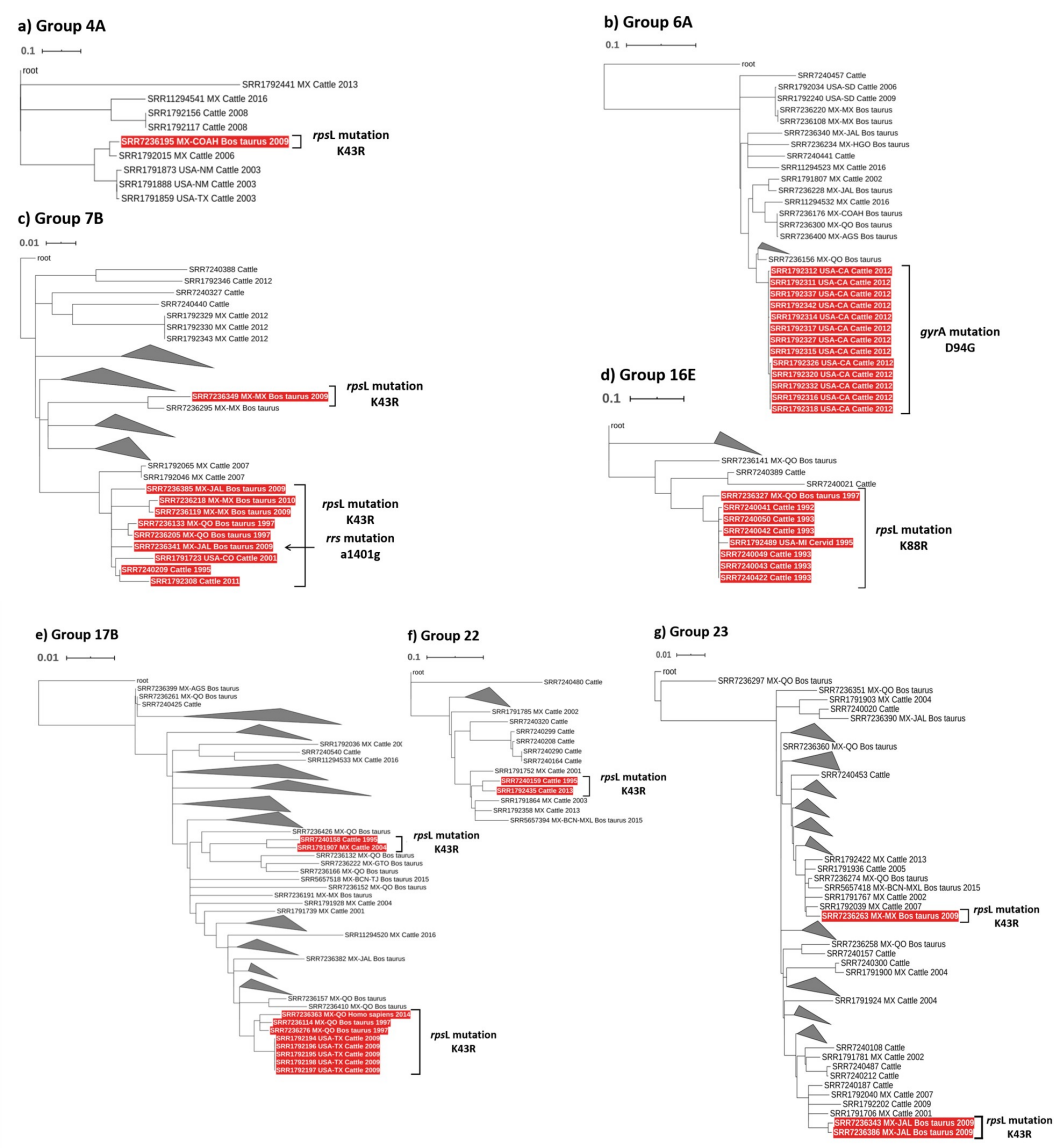
Phylogenetic groups 4A, 6A, 7B, 16E, 17B, 22 and 23 of *M*. *bovis*. a) Phylogram of group 4A showing 1 strain with a mutation of high confidence in the *rps*L K43R (Lys^43^Arg) gene that confers resistance to streptomycin. b) Phylogram of group 6A showing 13 strains with high confidence mutations in the *gyr*A (Asp^94^Gly) gene that confers resistance to fluoroquinolones. c) Phylogram of group 7B showing 10 strains with high confidence mutations in the *rps*L K43R (Lys^43^Arg) gene that confers resistance to streptomycin. SRR7236341 presents another mutation, g1401c, in the *rrs* gene, related to confer resistance to aminoglycosides. d) Phylogram of group 16E showing 8 strains with high confidence mutations in the *rps*L (Lys^88^Arg) gene. e) Phylogram of group 17B showing 10 strains with high confidence mutations in the *rps*L K43R (Lys^43^Arg) gene. f) Phylogram of group 22 showing 2 strains with the same high confidence mutations in the *rps*L (Lys^43^Arg) gene. g) Phylogram of group 23 showing 3 strains with high confidence mutations in the *rps*L K43R (Lys^43^Arg).

All isolates were subjected to phylogenetic analysis to assess their genetic relatedness. Thirty-two distinctive phylogenetic groups were identified, including 110 subgroups. These groups have been previously reported [25]. Two isolates, SRR7240297 and SRR7240428, harboring the S315T Ser^315^Thr mutation in the *kat*G gene were clustered together in group 10 (indistinguishable from each other). Twenty-six isolates harboring resistance-associated mutations K43R (Lys^43^Arg) in the *rps*L gene were clustered in phylogenetic groups 4A, 7B, 17B, 22 and 23 (Fig 1). Among all ten isolates included in group 7; all but one was genetically close SRR1791723, SRR1792308, SRR7236119, SRR7236133, SRR7236205, SRR7236218, SRR7236341, SRR7236385 and SRR7240209 with the exemption of strain SRR7236349. Six strains were originally from three different states in Mexico. The strains were recovered from the late 1990s until 2011. Similarly, eight strains bearing the mutation K43R (Lys^43^Arg) in the *rps*L gene and comprised within group 17B were genetically close (SRR1792194, SRR1792195, SRR1792196, SRR1792197, SRR1792198, SRR7236114, SRR7236276, and SRR7236363). Among these eight strains, five isolates showed identical sequences, collection year (2009) and geographical location (TX). The remaining three genetically close strains were recovered from Mexico, including one human case. All strains were collected at very different times. Additionally, two distant strains from group 17 (SRR7240158 and SRR1791907) were also identified. Two somehow close strain pairs were observed in groups 22 and 23. The pair in group 22 was more distant and isolates were collected 18 years apart. However, the pair in group 23 was significantly closer and both strains were recovered from Jalisco State in Mexico in 2009. The remaining strain in group 23, from a different state in Mexico, was significantly different from the isolates recovered from Jalisco State. Only one strain, SRR7236195, displaying the K43R (Lys^43^Arg) mutation was observed in group 4.

Eight strains (SRR7240041, SRR7240042, SRR7240043, SRR7240049, SRR7240050 and SRR7240422) were clustered into group 16E. Data on origin was not available for most of these isolates; however, the year of collection ranged between 1992 and 1997. Two strains, SRR7236327 and SRR1792489, were isolated from Queretaro State in Mexico and Michigan in 1995 and 1997, respectively.

All thirteen isolates (SRR1792311, SRR1792312, SRR1792314, SRR1792315, SRR1792316, SRR1792317, SRR1792318, SRR1792320, SRR1792326, SRR1792327, SRR1792332, SRR1792337, and SRR1792342) containing the *gyr*A D94G, Asp^94^Gly, resistance mutation were all comprised in group 6A. These isolates were closely genetically related and originally recovered from California in 2012 (Fig 1B). Finally, the aforementioned SRR7236341 strain, poly-resistant strain, was included in group 7.

## Discussion

Here, we have shown that *M*. *bovis* strains isolated from bovines harbored resistance-related mutations for first- and second-line antibiotics. Resistance to isoniazid, fluoroquinolones and aminoglycosides are particularly worrisome considering the risk for emergence of MDR and XDR TB. As far as we know, this is the first comprehensive study aiming to identify the drug resistance mutation patterns occurring among *M*. *bovis* in non-human cases. The main limitation in our study is the inclusion of strains recovered only from the Americas. This is a small sampling considering the prevalence of tuberculosis in many regions of the world. Therefore, inclusion of strains from other parts of the world is likely to increase the number of strains displaying drug resistance. Sequencing of *M*. *bovis* strains recovered from non-human hosts is infrequent. As a result, there is a significant underreporting of drug-related mutations among livestock and wildlife animals [26]. Indeed, several reports of *M*. *bovis* drug-resistant isolates recovered from humans, ranging from mono-, poly-resistant and MDR detected strains have been reported [2,27,28].

High confidence mutations conferring resistance to isoniazid, streptomycin, aminoglycosides, and fluoroquinolones identified in this study include the *kat*G S315T, *rps*L K43R and *rps*L K88R, *rrs* a1401g and *gyr*A D94G (Table 1). Drug resistance among *M*. *bovis* isolates have been previously reported, among strains recovered from cattle [29] and humans [30,31,27,28,9,32]. The occurrence and subsequently fixation of drug resistance conferring mutations is a major concern that jeopardizes the success of local tuberculosis control programs in the region. The use of antibiotics in food intended to feed livestock as additives to promote growth has been extensively reported [33,34]. The impact on human health of such practices have been a matter of concern due to the possibility of facilitating the occurrence of drug resistance strains [35]. However, first and second line antibiotics against TB are not approved for animal consumption. This further complicates the identification of the sources implicated in the acquisition of mutations conferring resistance to these drugs since multiple factors could participate in this process.

Isolates SRR7240297 and SRR7240428 harboring the S315T, Ser315Thr mutation in the *kat*G gene were isolated from cattle in the same year (1994) and belonged to the same phylogenetic group. S315T is considered as a “moderate-level” resistance and accounts for 95% of all *kat*G mutations [36]. Specific mutation patterns among *M*. *bovis* strains conferring resistance to isoniazid resistance, including the *kat*G S315T mutation recovered from cattle have been previously reported [29,37]. However, the mechanisms exploited by *M*. *bovis* to develop and fixate mutations conferring resistance are not well understood.

The *rps*L gene contains bona fide mutations conferring resistance to streptomycin. Resistant strains harboring K43R (Lys43Arg) and K88R (Lys88Arg) mutations, both widely reported in the literature as resistance markers [23,38]. Among these isolates, five strains were collected from dairy cows in Texas (2009). All these five isolates were closely genetically related (group 17), and likely belonged to the same transmission network (Fig 1E). Strains exhibiting drug resistance mutations clustered in group 7 were genetically closed (Fig 1C). Despite the lack of epidemiological relatedness among these isolates, the strains were significantly genetically close, highlighting the clonality of *M*. *tuberculosis*.

Regarding the double mutant strain, SRR7236341, for streptomycin and aminoglycosides simultaneous resistance, this could be considered rare and we found no previous reports of this double mutation. However, monoresistance for streptomycin in humans is not common, ranging from 0.9 to 5.7% [39]. Additionally, this double-mutant could be originated by the mechanism proposed by Reeves et al, mainly due to mutation of *whi*B7 untranslated region [40].

The increasing use of quinolones for the treatment of respiratory infectious diseases has led to the occurrence of quinolone resistant *M*. *tuberculosis* [41–43]. Residues S83 and D87 (equivalent to A90 and D94 in *M*. *tuberculosis gyr*A) are commonly mutated in quinolone-resistant strains, and both residues lie in the α4 helix of the helix-turn-helix region. S83A mutation results in low-level drug resistance; while modifications to bulky hydrophobic side chains, such as leucine, valine, phenylalanine, or tyrosine, confers high-level resistance [44,45,43]. Here, we identified thirteen strains harboring resistant mutation D94G in the *gyr*A gene. This mutation is associated with a high level of resistance to fluoroquinolones, which in consequence provides resistance to levofloxacin and moxifloxacin [46]. All thirteen isolates were genetically related and likely belonged to the same transmission network (group 6A). The use of fluoroquinolones in food animals has been previously reported [47]. As a consequence of their use, resistance in other bacterial genuses such as *Meinnheimia haemolytica* has also been reported in cattle [48,49]. The mechanisms exploited by *M*. *bovis* to acquire resistance to fluoroquinolones and aminoglycosides remain to be identified.

The identification of drug resistant *M*. *bovis* strains recovered from cattle to first- and second-line antibiotics, is of critical importance and jeopardizes the efforts to control spread of highly pathogenic mycobacteria. While use of first- and second-line antibiotics have been reported in cattle, the use of these anti-tuberculosis drugs in animals for human consumption is not recommended and infrequent. Thus, the occurrence of drug resistant mutants among cattle is likely to occur by anthroponotic (reverse zoonosis) transmission from humans carrying resistant strains. Indeed, anthroponotic transmission has been previously reported [50]. However, several studies in humans suggest that *M*. *bovis* zoonotic infection it is more likely to be caused by consumption of unpasteurized dairy products and less frequently attributed animal-to-human or human-to-human transmission [28,31,32].

Recently, it has been proposed that *M*.*bovis* transmission occurs from domesticated ruminants to humans and other primates in areas where this dynamic interface between different hosts and *M*. *bovis* takes place. This highlights the importance of advanced molecular characterization on identifying regions of high exposure, routes of transmission and phylogenetic relationships [51]. Importantly, the mechanisms exploited by *M*. *bovis* to develop and fixate mutations conferring resistance warrants further research.

## Conclusions

Our results showed mutations conferring drug resistance to first- and second-line antibiotics in different *M*. *bovis* lineages. These findings highlight the importance of implementing robust molecular surveillance of *M*. *bovis* lineages, both in humans and cattle, to monitor the emergence of mutants conferring drug resistance. The circulation of drug resistance strains in cattle represents a major risk for the occurrence of extensively resistant strains in the population. Implementation of advanced characterization of tuberculosis isolates will aid in the understanding of the transmission dynamics exploited by members of the tuberculosis complex among and between animals and humans.

## Supporting information

S1 Table Metadata of 2074 isolates of *Mycobacterium bovis*. (CSV)
