# Correction: Identification of drug resistance mutations among *Mycobacterium bovis* lineages in the Americas

**DOI:** 10.1371/journal.pntd.0011076

**Published:** 2023-01-24

**Authors:** Carlos Arturo Vázquez-Chacón, Felipe de Jesús Rodríguez-Gaxiola, Cruz Fernando López-Carrera, Mayra Cruz-Rivera, Armando Martínez-Guarneros, Ricardo Parra-Unda, Eliakym Arámbula-Meraz, Salvador Fonseca-Coronado, Gilberto Vaughan, Paúl Alexis López-Durán

There are errors in the affiliations for authors Carlos Arturo Vázquez-Chacón and Paúl Alexis López-Durán. The correct affiliations are as follows:

Carlos Arturo Vázquez-Chacón^1,2,3^, Felipe de Jesús Rodríguez-Gaxiola^4^, Cruz Fernando López-Carrera^5^, Mayra Cruz-Rivera^5^, Armando Martínez-Guarneros^2^, Ricardo Parra-Unda^6^, Eliakym Arámbula-Meraz^7^, Salvador Fonseca-Coronado^3^, Gilberto Vaughan^1^, Paúl Alexis López-Durán^1,4,5^

**1** Facultad de Ciencias de la Salud, Universidad Anáhuac, Campus Norte, Estado de México, México, **2** Facultad de Medicina y Cirugía, Universidad Autónoma Benito Juárez de Oaxaca, Oaxaca, México, **3** Laboratorio de Micobacterias, Instituto de Diagnóstico y Referencia Epidemiológicos, Ciudad de México, México, **4** Facultad de Estudios Superiores Cuautitlán, Universidad Nacional Autónoma de México, Estado de México, México, **5** Escuela Nacional de Ciencias Biológicas, Instituto Politécnico Nacional, **6** Facultad de Medicina, Universidad Nacional Autónoma de México, Ciudad de México, México, **7** Unidad de Investigaciones en Salud Pública, Facultad de Ciencias Químico Biológicas, Universidad Autónoma de Sinaloa, Culiacán, Sinaloa, México, **8** Laboratorio de Genética y Biología Molecular, Facultad de Ciencias Químico Biológicas, Universidad Autónoma de Sinaloa, Culiacán, Sinaloa, México.
